# A polymorph of *N*′-[(4-hy­droxy­phen­yl)methyl­idene]pyridine-4-carbohydrazide

**DOI:** 10.1107/S2414314626003147

**Published:** 2026-03-27

**Authors:** Lincy Tom, Jerry P. Jasinski, Victoria A. Smolenski, M. R. Prathapachandra Kurup

**Affiliations:** a Department of Chemistry, Nirmala College Muvattupuzha, Ernakulam, 686661, Kerala, India; b Department of Chemistry Keene, State College, 229 Main Street Keene NH03435-2001 USA; c Department of Chemistry Central University of Kerala, Tejaswini Hills Periye Kasaragod 671 320 Kerala India; Vienna University of Technology, Austria

**Keywords:** isoniazid, crystal structure, hydrogen bonding, packing polymorphism, Hirshfeld

## Abstract

The synthesis and single-crystal X-ray diffraction analysis of a polymorphic form of the isoniazid derivative *N*′-[(4-hy­droxy­phen­yl)methyl­idene]pyridine-4-carbohydrazide, C_13_H_11_N_3_O_2_, are reported, revealing that both the mol­ecular conformation and the hydrogen-bonding scheme differ in the two polymorphs. The crystal packing of the title polymorph is primarily consolidated by N—H⋯O and O—H⋯N hydrogen-bonding inter­actions, leading to the formation of a supra­molecular layer parallel to (001). The inter­molecular contacts were further qu­anti­fied and analysed using Hirshfeld surface analysis.

## Abstract 2

The crystal structure of a polymorph of *N*′-[(4-hy­droxy­phen­yl)methyl­idene]pyridine-4-carbohydrazide reveals a distinct supra­molecular arrangement arising from variations in inter­molecular hydrogen bonding relative to the known polymorph.

## Graphical abstract

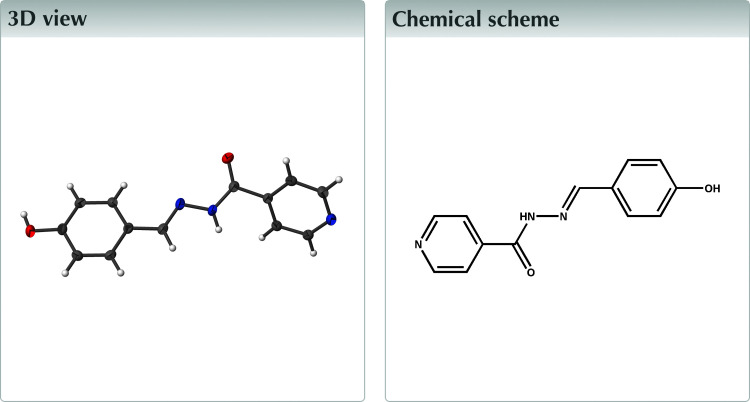

## Structure description

N-containing heterocycles constitute a major class of natural products and possess a wide range of applications (Goetz *et al.*, 2015 ▸). They are widely used as starting materials for the synthesis of biologically important compounds. Among them, isoniazid-based scaffolds, which contain nitro­gen heterocycles, have attracted considerable attention in medicinal chemistry due to their diverse biological activities, including anti-carcinogenic, anti-fungal, anti-mycobacterial, analgesic, anti­bacterial, and anti­viral properties (Tom *et al.*, 2020 ▸; Rodrigues *et al.*, 2014 ▸; Mohanram & Meshram, 2014 ▸; Judge *et al.*, 2013 ▸; Hu *et al.*, 2017 ▸; Costa *et al.*, 2024 ▸). Among these, their anti-tubercular activity is particularly significant. As part of our ongoing research in this field, we report here on the synthesis and crystal structure of a polymorph of *N*′-[(4-hy­droxy­phen­yl)methyl­idene]pyridine-4-carbohydrazide, an isoniazid-derived mol­ecule with potential anti­tubercular activity.

**Figure 1:**
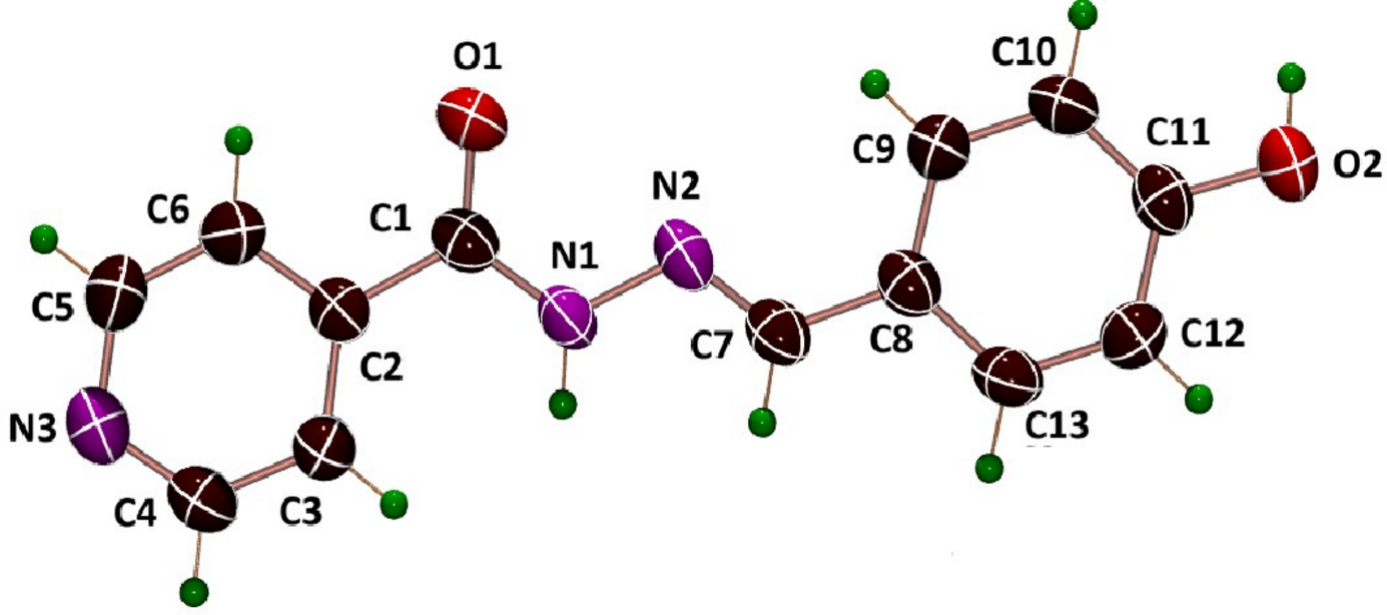
Mol­ecular structure of polymorph *P*_A_ with displacement ellipsoids drawn at the 30% probability level.

**Figure 2:**
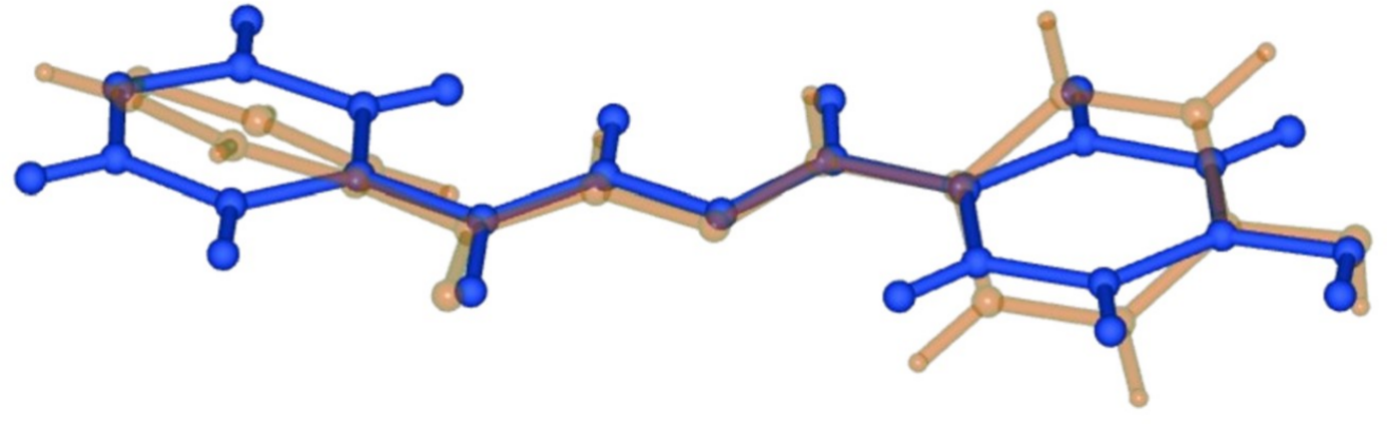
Superimposition of the mol­ecular structures of *P*_A_ (gold) and *P*_B_ (blue).

Single-crystal X-ray diffraction analysis of the title compound, C_13_H_11_N_3_O_2_, revealed that it is dimorphic. The title *P*_A_ polymorph (Fig. 1 ▸) crystallizes in the monoclinic space group *P*2_1_/*c*. The previously reported polymorph *P*_B_ (Deng *et al.*, 2005 ▸) crystallizes in the same space group type but with different unit-cell parameters. The differences between the *P*_A_ and *P*_B_ forms can mainly be attributed to crystal-packing effects. An additional monohydrated ortho­rhom­bic form has also been reported (Tai *et al.*, 2007 ▸). The overlay of the two mol­ecules of the polymorphs (Fig. 2 ▸) indicates that they possess distinct mol­ecular conformations. *P*_A_ adopts a slightly twisted conformation compared to the more planar *P*_B_ form. The dihedral angles between the pyridine ring (C2, C3, C4, N3, C5, C6) and hydrazide moiety (N1, N2, C1, O1, C7) are 36.14 (8)° in *P*_A_ and 9.59 (8)° in *P*_B_. Similarly, the dihedral angles between the hydrazide moiety and phenol ring are 23.97 (8)° in *P*_A_ and 3.96 (7)° in *P*_B_.

**Figure 3:**
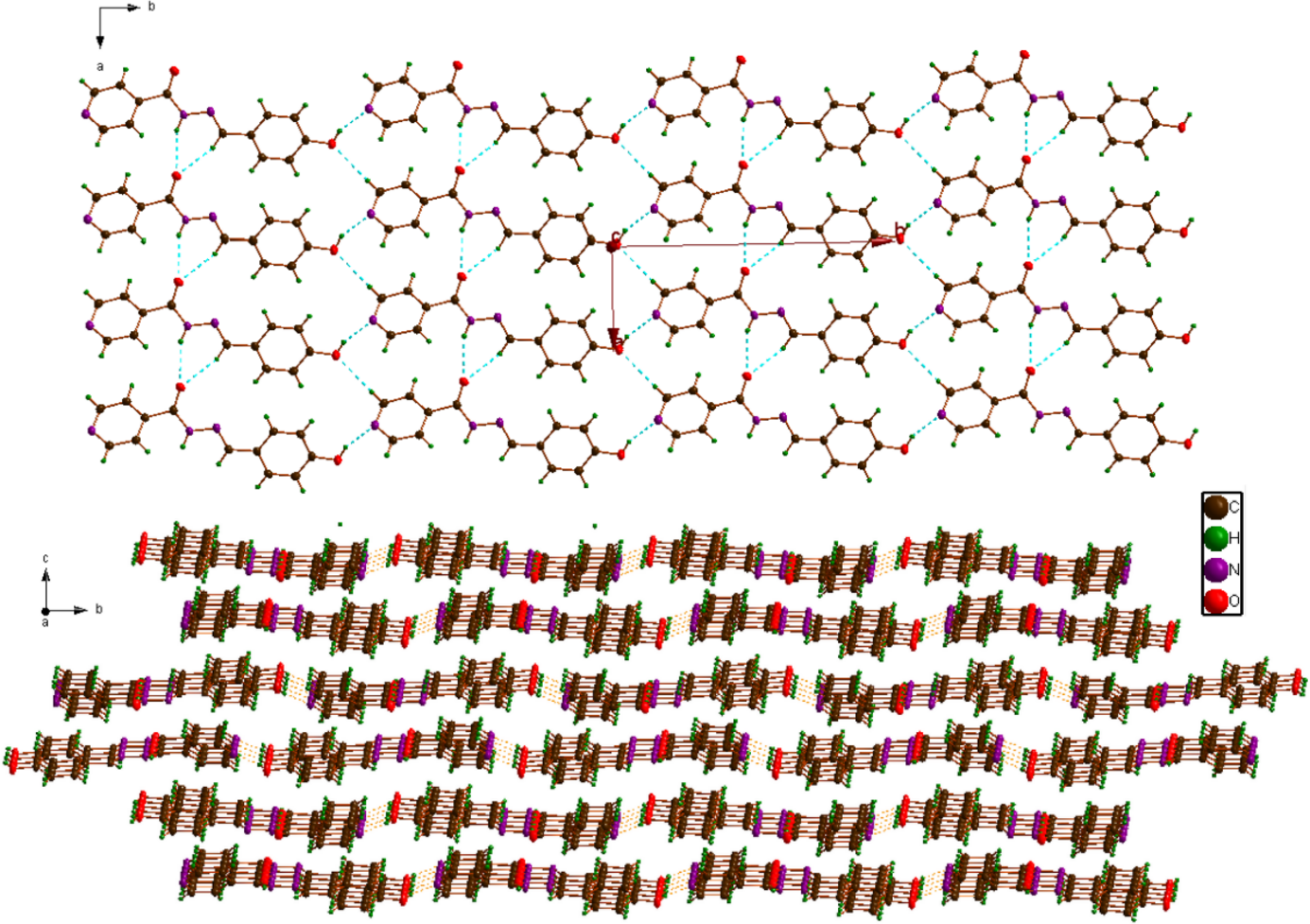
Formation of supra­molecular (001) layers in the crystal structure of *P*_A_. Hydrogen bonds are shown as dotted lines.

The mol­ecular packing and hydrogen-bonding patterns differ significantly in the two extended structures of *P*_A_ and *P*_B_. The polymorphs exhibit different combinations of hydrogen-bonding donor and acceptor sites, leading to distinct supra­molecular networks. The *P*_A_ polymorph reported here exhibits two types of classical hydrogen-bonding inter­actions (Table 1 ▸). The NH group of the N=NH—C=O moiety is hydrogen-bonded to the O atom of the same moiety of a neighbouring mol­ecule into chains extending parallel to [100]. These chains are connected through O—H⋯N hydrogen-bonding inter­actions involving the phenol OH group and the pyridine N atom, leading to the formation of a supra­molecular layer extending parallel to (001) (Fig. 3 ▸). In polymorph *P*_B_, the NH group of the N=NH—C=O moiety bonds to the pyridine N atom, and the phenol OH group forms bifurcated hydrogen bonds to the N=NH—C=O moiety and the carbonyl O atom of neighbouring mol­ecules, thus leading to a different supra­molecular arrangement.

**Figure 4:**
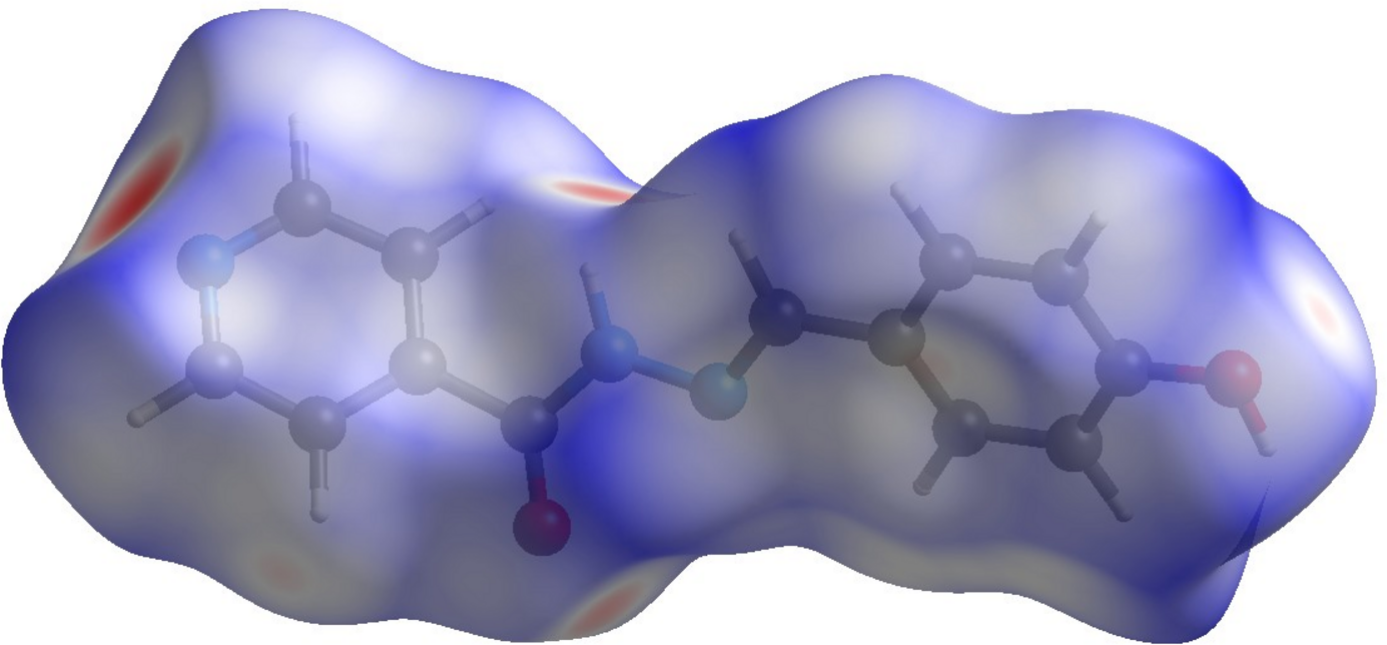
The Hirshfeld surface of *P*_A_ mapped over *d*_norm_.

**Figure 5:**
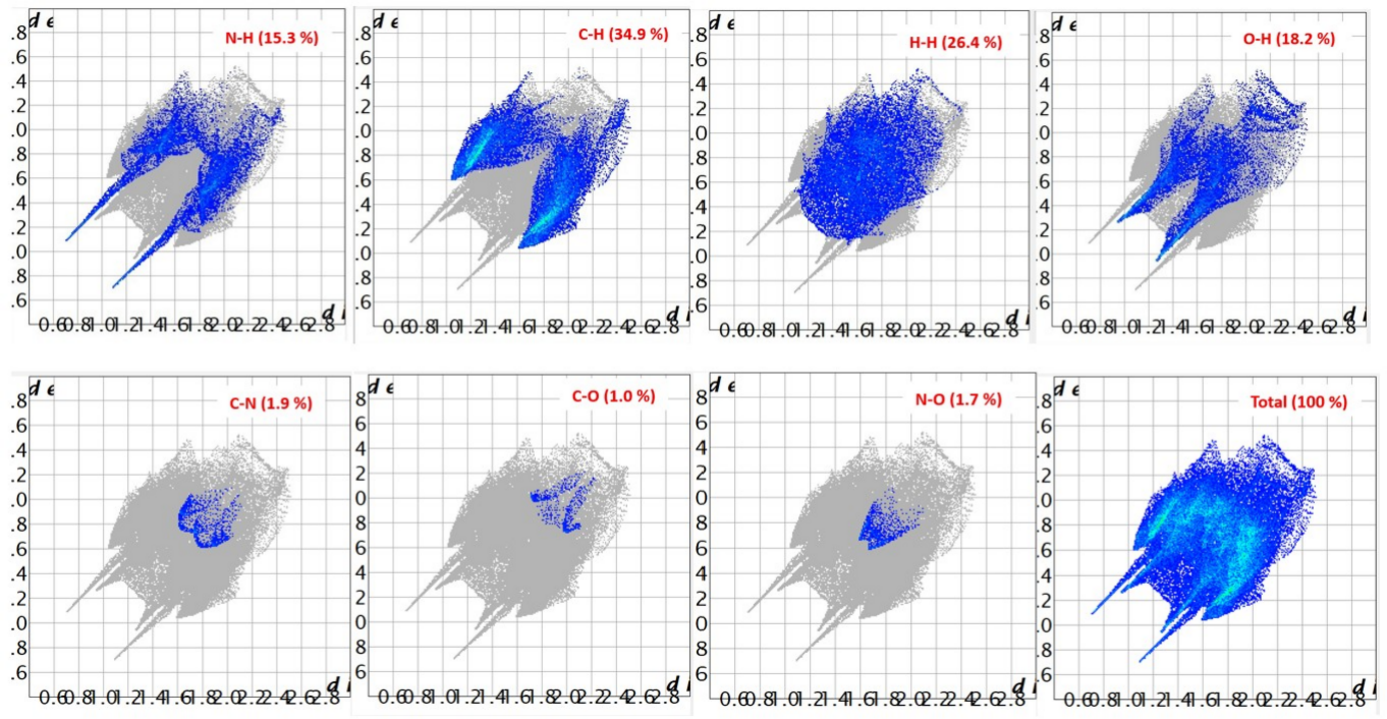
The two-dimensional fingerprint plots for *P*_A_ for different contact types.

To visualize and qu­antify the contributions of inter­molecular inter­actions in the supra­molecular assembly of the title compound, a Hirshfeld surface (HS) analysis (Spackman & Jayatilaka, 2009 ▸) was performed using *CrystalExplorer* (Spackman *et al.*, 2021 ▸). The Hirshfeld surface mapped over *d*_norm_ is shown in Fig. 4 ▸, where prominent deep-red spots correspond to significant inter­molecular contacts. These spots appear around atoms N3, N1, O1 and O2, indicating that these atoms participate in the inter­molecular hydrogen bonds, as discussed above. The associated two-dimensional fingerprint plots provide qu­anti­tative insight into the various non-covalent inter­actions contributing to the crystal packing. The H⋯H, C⋯H, N⋯H and O⋯H, inter­actions dominate the packing, collectively accounting for approximately 95% of the total Hirshfeld surface area (Fig. 5 ▸).

## Synthesis and crystallization

A mixture of *p*-hy­droxy­benzaldehyde (1 mmol) and isonicotinic hydrazide (1 mmol) was refluxed in methanol (20 ml) at 343 K for 30 min. After completion of the reaction, the mixture was allowed to cool to room temperature. Colourless crystals suitable for X-ray diffraction were obtained by slow evaporation of the solution at room temperature.

## Refinement

Crystal data, data collection and structure refinement details are summarized in Table 2 ▸.

## Supplementary Material

Crystal structure: contains datablock(s) I. DOI: 10.1107/S2414314626003147/wm4248sup1.cif

Structure factors: contains datablock(s) I. DOI: 10.1107/S2414314626003147/wm4248Isup2.hkl

Supporting information file. DOI: 10.1107/S2414314626003147/wm4248Isup3.cml

CCDC reference: 2540578

Additional supporting information:  crystallographic information; 3D view; checkCIF report

## Figures and Tables

**Table 1:** Hydrogen-bond geometry (Å, °)

*D*—H⋯*A*	*D*—H	H⋯*A*	*D*⋯*A*	*D*—H⋯*A*
N1—H1⋯O1^i^	0.86	2.34	3.1452 (15)	156
O2—H2⋯N3^ii^	0.82	1.96	2.7356 (16)	159

Symmetry codes: (i) 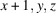; (ii) 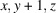.

**Table 2:** Experimental details

Crystal data	
Chemical formula	C_13_H_11_N_3_O_2_
*M*_r_	241.25
Crystal system, space group	Monoclinic, *P*2_1_/*c*
Temperature (K)	293
*a*, *b*, *c* (Å)	5.36340 (13), 14.4462 (4), 14.6619 (4)
β (°)	91.229 (3)
*V* (Å^3^)	1135.75 (5)
*Z*	4
Radiation type	Cu *K*α
μ (mm^−1^)	0.81
Crystal size (mm)	0.42 × 0.33 × 0.29

Data collection	
Diffractometer	Rigaku Oxford Diffraction Gemini Eos
Absorption correction	Multi-scan (*CrysAlis PRO*; Rigaku OD, 2015 ▸)
*T*_min_, *T*_max_	0.858, 1.000
No. of measured, independent and observed [*I* > 2σ(*I*)] reflections	7898, 2185, 1901
*R*_int_	0.027
(sin θ/λ)_max_ (Å^−1^)	0.624

Refinement	
*R*[*F*^2^ > 2σ(*F*^2^)], *wR*(*F*^2^), *S*	0.039, 0.112, 1.05
No. of reflections	2185
No. of parameters	164
H-atom treatment	H-atom parameters constrained
Δρ_max_, Δρ_min_ (e Å^−3^)	0.22, −0.25

Computer programs: *CrysAlis PRO* (Rigaku OD, 2015 ▸), *SHELXT* (Sheldrick, 2015*a* ▸), *SHELXL* (Sheldrick, 2015*b* ▸), *ORTEP-3 for Windows* (Farrugia, 2012 ▸), *Mercury* (Macrae *et al.*, 2020 ▸) and *publCIF* (Westrip, 2010 ▸).

## full crystallographic data

### N'-[(4-Hydroxyphenyl)methylidene]pyridine-4-carbohydrazide Crystal data

**Table:**

C_13_H_11_N_3_O_2_	*F*(000) = 504
*M**_r_* = 241.25	*D*_x_ = 1.411 Mg m^−^^3^
Monoclinic, *P*2_1_/*c*	Cu *K*α radiation, λ = 1.54184 Å
*a* = 5.36340 (13) Å	Cell parameters from 3210 reflections
*b* = 14.4462 (4) Å	θ = 4.3–71.3°
*c* = 14.6619 (4) Å	µ = 0.81 mm^−^^1^
β = 91.229 (3)°	*T* = 293 K
*V* = 1135.75 (5) Å^3^	Block, pale yellow
*Z* = 4	0.42 × 0.33 × 0.29 mm

### N'-[(4-Hydroxyphenyl)methylidene]pyridine-4-carbohydrazide Data collection

**Table:**

Rigaku Oxford Diffraction Gemini Eos diffractometer	1901 reflections with *I* > 2σ(*I*)
Radiation source: Enhance (Cu) X-ray Source	*R*_int_ = 0.027
ω scans	θ_max_ = 74.3°, θ_min_ = 4.3°
Absorption correction: multi-scan (CrysAlisPro; Rigaku OD, 2015)	*h* = −6→5
*T*_min_ = 0.858, *T*_max_ = 1.000	*k* = −17→17
7898 measured reflections	*l* = −17→18
2185 independent reflections	

### N'-[(4-Hydroxyphenyl)methylidene]pyridine-4-carbohydrazide Refinement

**Table:**

Refinement on *F*^2^	0 restraints
Least-squares matrix: full	Hydrogen site location: inferred from neighbouring sites
*R*[*F*^2^ > 2σ(*F*^2^)] = 0.039	H-atom parameters constrained
*wR*(*F*^2^) = 0.112	*w* = 1/[σ^2^(*F*_o_^2^) + (0.0635*P*)^2^ + 0.3435*P*] where *P* = (*F*_o_^2^ + 2*F*_c_^2^)/3
*S* = 1.05	(Δ/σ)_max_ = 0.001
2185 reflections	Δρ_max_ = 0.22 e Å^−^^3^
164 parameters	Δρ_min_ = −0.25 e Å^−^^3^

### N'-[(4-Hydroxyphenyl)methylidene]pyridine-4-carbohydrazide Special details

**Table:**

Geometry. All esds (except the esd in the dihedral angle between two l.s. planes) are estimated using the full covariance matrix. The cell esds are taken into account individually in the estimation of esds in distances, angles and torsion angles; correlations between esds in cell parameters are only used when they are defined by crystal symmetry. An approximate (isotropic) treatment of cell esds is used for estimating esds involving l.s. planes.

### N'-[(4-Hydroxyphenyl)methylidene]pyridine-4-carbohydrazide Fractional atomic coordinates and isotropic or equivalent isotropic displacement parameters (Å^2^)

**Table:**

	*x*	*y*	*z*	*U*_iso_*/*U*_eq_	
C1	0.4746 (3)	0.44240 (9)	0.39335 (9)	0.0229 (3)	
C2	0.5399 (2)	0.34115 (9)	0.39757 (9)	0.0211 (3)	
C3	0.7526 (2)	0.30837 (9)	0.43964 (9)	0.0235 (3)	
H3	0.863180	0.348585	0.469094	0.028*	
C4	0.7997 (3)	0.21405 (10)	0.43747 (10)	0.0252 (3)	
H4	0.943707	0.192648	0.466990	0.030*	
C5	0.4466 (3)	0.18484 (10)	0.35751 (10)	0.0269 (3)	
H5	0.337940	0.143061	0.329277	0.032*	
C6	0.3834 (2)	0.27780 (10)	0.35686 (9)	0.0246 (3)	
H6	0.235113	0.297153	0.328877	0.029*	
C7	0.8580 (3)	0.63450 (9)	0.36248 (9)	0.0236 (3)	
H7	0.996667	0.598599	0.349456	0.028*	
C8	0.8798 (2)	0.73497 (9)	0.35866 (9)	0.0218 (3)	
C9	0.6961 (2)	0.79454 (10)	0.39373 (9)	0.0237 (3)	
H9	0.554751	0.769727	0.420074	0.028*	
C10	0.7254 (3)	0.88975 (10)	0.38910 (10)	0.0262 (3)	
H10	0.605271	0.928803	0.412908	0.031*	
C11	0.9400 (3)	0.92725 (10)	0.34775 (10)	0.0252 (3)	
C12	1.1243 (3)	0.86858 (10)	0.31337 (10)	0.0250 (3)	
H12	1.265131	0.893409	0.286667	0.030*	
C13	1.0954 (3)	0.77356 (10)	0.31942 (10)	0.0240 (3)	
H13	1.218714	0.734688	0.297531	0.029*	
N1	0.6758 (2)	0.49791 (8)	0.38618 (8)	0.0244 (3)	
H1	0.821556	0.473419	0.383314	0.029*	
N2	0.6528 (2)	0.59354 (8)	0.38331 (8)	0.0252 (3)	
N3	0.6540 (2)	0.15252 (8)	0.39630 (8)	0.0265 (3)	
O1	0.25700 (19)	0.46875 (7)	0.39380 (8)	0.0327 (3)	
O2	0.9794 (2)	1.01946 (7)	0.34003 (9)	0.0354 (3)	
H2	0.858366	1.047600	0.359096	0.053*	

### N'-[(4-Hydroxyphenyl)methylidene]pyridine-4-carbohydrazide Atomic displacement parameters (Å^2^)

**Table:**

	*U*^11^	*U*^22^	*U*^33^	*U*^12^	*U*^13^	*U*^23^
C1	0.0241 (7)	0.0206 (7)	0.0240 (7)	0.0022 (5)	−0.0011 (5)	−0.0009 (5)
C2	0.0210 (7)	0.0194 (7)	0.0230 (7)	0.0008 (5)	0.0024 (5)	0.0005 (5)
C3	0.0216 (7)	0.0219 (7)	0.0268 (7)	−0.0018 (5)	−0.0013 (5)	0.0003 (5)
C4	0.0217 (7)	0.0237 (7)	0.0301 (7)	0.0009 (5)	−0.0008 (6)	0.0024 (6)
C5	0.0259 (7)	0.0232 (7)	0.0315 (8)	−0.0046 (6)	−0.0012 (6)	−0.0026 (6)
C6	0.0198 (7)	0.0258 (7)	0.0280 (7)	−0.0008 (5)	−0.0018 (5)	0.0008 (5)
C7	0.0246 (7)	0.0200 (7)	0.0259 (7)	0.0038 (5)	−0.0027 (5)	−0.0017 (5)
C8	0.0230 (7)	0.0188 (7)	0.0233 (7)	0.0017 (5)	−0.0049 (5)	−0.0001 (5)
C9	0.0203 (6)	0.0212 (7)	0.0295 (7)	−0.0009 (5)	0.0006 (5)	0.0004 (5)
C10	0.0215 (7)	0.0238 (7)	0.0334 (7)	0.0039 (5)	0.0011 (6)	−0.0028 (6)
C11	0.0261 (7)	0.0186 (7)	0.0308 (7)	0.0002 (5)	−0.0040 (6)	0.0008 (5)
C12	0.0207 (6)	0.0254 (7)	0.0290 (7)	−0.0022 (5)	0.0018 (5)	0.0017 (6)
C13	0.0211 (7)	0.0237 (7)	0.0273 (7)	0.0049 (5)	−0.0008 (5)	−0.0027 (5)
N1	0.0238 (6)	0.0153 (6)	0.0340 (7)	0.0029 (4)	−0.0011 (5)	−0.0004 (5)
N2	0.0285 (6)	0.0166 (6)	0.0306 (6)	0.0017 (5)	−0.0021 (5)	−0.0013 (5)
N3	0.0269 (6)	0.0195 (6)	0.0332 (7)	0.0003 (5)	0.0028 (5)	0.0004 (5)
O1	0.0240 (5)	0.0257 (5)	0.0484 (7)	0.0053 (4)	−0.0031 (4)	0.0013 (5)
O2	0.0307 (6)	0.0174 (5)	0.0583 (8)	−0.0003 (4)	0.0063 (5)	0.0003 (5)

### N'-[(4-Hydroxyphenyl)methylidene]pyridine-4-carbohydrazide Geometric parameters (Å, º)

**Table:**

C1—O1	1.2279 (18)	C7—H7	0.9300
C1—N1	1.3501 (18)	C8—C9	1.4132 (19)
C1—C2	1.5049 (18)	C8—C13	1.417 (2)
C2—C3	1.3696 (19)	C9—C10	1.3862 (19)
C2—C6	1.3701 (19)	C9—H9	0.9300
C3—C4	1.3862 (19)	C10—C11	1.420 (2)
C3—H3	0.9300	C10—H10	0.9300
C4—N3	1.3212 (18)	C11—O2	1.3538 (17)
C4—H4	0.9300	C11—C12	1.404 (2)
C5—N3	1.3234 (18)	C12—C13	1.3844 (19)
C5—C6	1.385 (2)	C12—H12	0.9300
C5—H5	0.9300	C13—H13	0.9300
C6—H6	0.9300	N1—N2	1.3875 (16)
C7—N2	1.2916 (19)	N1—H1	0.8600
C7—C8	1.4573 (18)	O2—H2	0.8200
			
O1—C1—N1	125.28 (12)	C13—C8—C7	118.31 (12)
O1—C1—C2	121.43 (12)	C10—C9—C8	120.38 (13)
N1—C1—C2	113.26 (12)	C10—C9—H9	119.8
C3—C2—C6	117.50 (13)	C8—C9—H9	119.8
C3—C2—C1	123.05 (12)	C9—C10—C11	119.56 (13)
C6—C2—C1	119.44 (12)	C9—C10—H10	120.2
C2—C3—C4	118.69 (13)	C11—C10—H10	120.2
C2—C3—H3	120.7	O2—C11—C12	116.86 (13)
C4—C3—H3	120.7	O2—C11—C10	122.71 (13)
N3—C4—C3	124.43 (13)	C12—C11—C10	120.43 (13)
N3—C4—H4	117.8	C13—C12—C11	119.66 (13)
C3—C4—H4	117.8	C13—C12—H12	120.2
N3—C5—C6	123.26 (13)	C11—C12—H12	120.2
N3—C5—H5	118.4	C12—C13—C8	120.64 (13)
C6—C5—H5	118.4	C12—C13—H13	119.7
C2—C6—C5	119.81 (13)	C8—C13—H13	119.7
C2—C6—H6	120.1	C1—N1—N2	121.55 (11)
C5—C6—H6	120.1	C1—N1—H1	119.2
N2—C7—C8	122.33 (12)	N2—N1—H1	119.2
N2—C7—H7	118.8	C7—N2—N1	112.82 (11)
C8—C7—H7	118.8	C4—N3—C5	116.27 (12)
C9—C8—C13	119.31 (13)	C11—O2—H2	109.5
C9—C8—C7	122.37 (13)		
			
O1—C1—C2—C3	−147.48 (14)	C8—C9—C10—C11	0.8 (2)
N1—C1—C2—C3	34.59 (18)	C9—C10—C11—O2	179.30 (13)
O1—C1—C2—C6	33.4 (2)	C9—C10—C11—C12	−1.3 (2)
N1—C1—C2—C6	−144.56 (13)	O2—C11—C12—C13	179.82 (13)
C6—C2—C3—C4	1.1 (2)	C10—C11—C12—C13	0.4 (2)
C1—C2—C3—C4	−178.08 (13)	C11—C12—C13—C8	1.0 (2)
C2—C3—C4—N3	0.8 (2)	C9—C8—C13—C12	−1.50 (19)
C3—C2—C6—C5	−1.6 (2)	C7—C8—C13—C12	179.42 (12)
C1—C2—C6—C5	177.62 (13)	O1—C1—N1—N2	3.5 (2)
N3—C5—C6—C2	0.2 (2)	C2—C1—N1—N2	−178.65 (11)
N2—C7—C8—C9	12.3 (2)	C8—C7—N2—N1	−177.78 (11)
N2—C7—C8—C13	−168.67 (13)	C1—N1—N2—C7	−170.07 (12)
C13—C8—C9—C10	0.57 (19)	C3—C4—N3—C5	−2.1 (2)
C7—C8—C9—C10	179.61 (12)	C6—C5—N3—C4	1.6 (2)

### N'-[(4-Hydroxyphenyl)methylidene]pyridine-4-carbohydrazide Hydrogen-bond geometry (Å, º)

**Table:**

*D*—H···*A*	*D*—H	H···*A*	*D*···*A*	*D*—H···*A*
N1—H1···O1^i^	0.86	2.34	3.1452 (15)	156
O2—H2···N3^ii^	0.82	1.96	2.7356 (16)	159

Symmetry codes: (i) *x*+1, *y*, *z*; (ii) *x*, *y*+1, *z*.
